# Emerging *bla*_NDM_-positive *Salmonella enterica* in Chinese pediatric infections

**DOI:** 10.1128/spectrum.01485-24

**Published:** 2024-10-18

**Authors:** Yefang Ke, Zhe Zhu, Wenbo Lu, Wenyuan Liu, Lina Ye, Chenghao Jia, Min Yue

**Affiliations:** 1 Department of Clinical Laboratory, Women and Children’s Hospital of Ningbo University, Ningbo, China; 2 Ningbo Key Laboratory for the Prevention and Treatment of Embryogenic Diseases, Women and Children’s Hospital of Ningbo University, Ningbo, China; 3 Department of Blood Transfusion, Ningbo No. 2 Hospital, Ningbo, China; 4 Department of Veterinary Medicine, Zhejiang University College of Animal Sciences, Hangzhou, China; 5 School of Life Science, Hangzhou Institute for Advanced Study, University of Chinese Academy of Sciences, Hangzhou, China; University of Pittsburgh School of Medicine, Pittsburgh, Pennsylvania, USA

**Keywords:** *Salmonella enterica*, Typhimurium, carbapenem-resistant, NDM, phylogeography

## Abstract

Non-typhoidal *Salmonella* (NTS) is a common zoonotic foodborne pathogen, whose rising antimicrobial resistance has been an urgent threat to global public health. Here, we reported two carbapenem-resistant *Salmonella enterica* (CRSE) strains (NBFE-049 and NBFE-164) carrying the *bla*_NDM_ genes, which were recovered from two Chinese children, belonged to *Salmonella enterica* serovar Typhimurium (*S*. Typhimurium) monophasic variant (*S*. 1,4,[5],12:i:-) ST34 (a sequence type) and *S*. Typhimurium ST19, respectively. Genes *bla*_NDM-5_ and *bla*_NDM-13_ were detected in NBFE-049 and NBFE-164, respectively. The *bla*_NDM-5_ in NBFE-049 was located in an IncHI2-type plasmid, named pNBFE-049. In NBFE-164, the *bla*_NDM-13_ was located in an IncI1-type plasmid, named pNBFE-164. The plasmid pNBFE-164 successfully transferred its resistance phenotype into the recipient strain *Escherichia coli* J53 with a high efficiency of 1.1 × 10^−2^, while no transconjugants were obtained in pNBFE-049 conjugation assays. We further elucidated the genetic relationships of globally occurring New Delhi Metallo-β-lactamase (NDM)-positive strains and locally distributed clinical strains within the same serovar. The closest relative of NBFE-049 was clinical *Salmonella* strain 1722, which was recovered in 2020 and differed by only three Single Nucleotide Polymorphisms (SNPs). No NDM-positive ST19 could be found in the National Center for Biotechnology Information (NCBI) database, and NBFE-164 showed a close genetic relationship with the other ST19 in this area. To sum up, we suggested the potential contributions of clonal spread and plasmid-mediated *bla*_NDM_ transfer in CRSE dissemination. This study reported the complete genome of two *bla*_NDM_-carrying *S*. Typhimurium isolates, shedding new insights into the antimicrobial resistance mechanisms and dissemination patterns of the emerging CRSE.

**IMPORTANCE:**

NTS is one of the most common zoonotic pathogens that causes foodborne illnesses, while *S*. Typhimurium is one of the most common serovars. With the rising prevalence of multi-resistant *Salmonella* worldwide, carbapenems have emerged as the last-line antibiotics for treating severe bacterial infections. In this study, we reported the genomic characteristics of two carbapenem-resistant *S*. Typhimurium strains, which were recovered from two pediatric patients, carrying *bla*_NDM-5_ and *bla*_NDM-13_, providing new insights into the antimicrobial resistance deteriminants and transmission risk of *bla*_NDM_-positive NTS in China. We suggested the potential contributions of clonal spread and plasmid-mediated *bla*_NDM_ transfer in CRSE dissemination. Future enhanced surveillance policy should mitigate CRSE spreading, and more importantly, clinical antimicrobial therapeutic regimens should be adjusted accordingly.

## INTRODUCTION

Non-typhoidal *Salmonella* (NTS) is one of the most common zoonotic pathogens that causes foodborne illnesses with a significant burden worldwide, including China (1–4). There are more than 2,600 serovars of NTS, while *S*. Typhimurium is one of the most common serovars (5).

NTS usually causes self-limiting diarrhea but may also lead to severe infections, frequently named invasive NTS diseases (6–12), which often require antibiotic prescriptions. With the rising prevalence of multi-resistant *Salmonella* worldwide, carbapenems have emerged as the last-line antibiotics for treating severe infections (13, 14). Children, particularly susceptible to NTS infection, are usually limited by the choice of antimicrobial therapy (9, 15).

Nevertheless, with the widespread usage in clinical and veterinary care (16, 17), increasing Carbapenem-resistant Enterobacteriaceae emerged, posing an urgent threat to public health (13, 18). Producing carbapenemases is the essential mechanism of carbapenem resistance, of which New Delhi Metallo-β-lactamase (NDM) was the most common carbapenemase in clinical practice in China (19). Carbapenem-resistant *Salmonella enterica* (*S. enterica*, [CRSE]) was reported in sporadic cases (19–22). However, considering NTS is a foodborne pathogen, a One Health approach for CRSE investigation remains unaddressed (23).

To improve our understanding of the genetic determinants and transmission risks of CRSE, we reported two isolates of NDM-producing NTS, which harbored *bla*_NDM-5_, and *bla*_NDM-13_, respectively, recovered from the feces of two Chinese children.

## RESULTS

### Antimicrobial resistance phenotypes

The *Salmonella* NBFE-049 and NBFE-164 strains were isolated from the fecal samples of two children. Generally, minimum inhibitory concentrations (MICs) of the NBFE-049 strain to ertapenem and imipenem were ≥8 µg/mL and 4 µg/mL, respectively. MICs of the NBFE-164 strain to ertapenem and imipenem were ≥8 µg/mL and ≥16 µg/mL, respectively. Besides, the NBFE-049 strain was resistant to most tested β-lactams and penicillin/β-lactamase-inhibitor, i.e., imipenem, ertapenem, ampicillin, ampicillin/sulbactam (SAM), ceftriaxone, cefepime, ceftazidime, and piperacillin/tazobactam (TZP), while susceptible to ciprofloxacin, levofloxacin, trimethoprim/sulfamethoxazole (TMP-SMX), aztreonam, and azithromycin. On the other hand, the NBFE-164 strain was non-susceptible to all tested antibiotics except for aztreonam and azithromycin (Table 1).

**TABLE 1:** MIC values of antimicrobials for NBFE-049, NBFE-164, and NBFE-164’s transconjugant^*a*^

Antimicrobial agent	NBFE-049		NBFE-164		J53		NBFE-164-J53	
	MIC (μg/mL)	Interpretation	MIC (μg/mL)	Interpretation	MIC (μg/mL)	Interpretation	MIC (μg/mL)	Interpretation
Imipenem	4	R^*b*^	≥16	R	≤1	S^*d*^	≥16	R
Ertapenem	≥8	R	≥8	R	≤0.5	S	≥8	R
Ampicillin	≥32	R	≥32	R	8	S	≥32	R
SAM	≥32	R	≥32	R	≤2	S	≥32	R
Ceftriaxone	≥64	R	≥64	R	≤1	S	≥64	R
Cefepime	≥64	R	32	R	≤1	S	8	I^*c*^
Ceftazidime	≥64	R	≥64	R	≤1	S	≥64	R
Ciprofloxacin	≤0.25	S	1	R	≤0.25	S	≤0.25	S
Levofloxacin	≤0.25	S	1	I	≤0.25	S	≤0.25	S
TMP-SMX	≤1/19	S	≥16/304	R	≤20	S	≤20	S
Aztreonam	≤1	S	≤1	S	≤1	S	≤1	S
TZP	≥128	R	≥128	R	≤4	S	≥128	R
Azithromycin	8	S	8	S	4	-	4	-

^*a*^ SAM, ampicillin/sulbactam; TMP-SMX, trimethoprim/sulfamethoxazole; and TZP, piperacillin/tazobactam.

^*b*^ R, Resistant.

^*c*^ I, Intermediate.

^*d*^ S, Susceptiable.

### Whole-genome sequencing and antimicrobial-resistant determinants

It was found that the NBFE-049 strain belongs to the *S*. Typhimurium monophasic variant (*S*. 1,4,[5],12:i:-) ST34 (sequence type), while the NBFE-164 strain was identified as *S*. Typhimurium ST19. Antimicrobial resistance genes (ARGs) *bla*_NDM-5_ and *bla*_NDM-13_ were detected in the NBFE-049 and NBFE-164 strains, respectively. The NBFE-049 strain also carried other ARGs, including *aac(6')-Iaa*, *aac(6')-Ib-cr*, *aph(3'')-Ib*, and *aph(6)-Id* (aminoglycoside resistance), *bla*_OXA-1_ (β-lactams), *catB3* (phenicol), *sul1* and *sul2* (sulfonamide), *tet(B)* (tetracycline), and *ARR-3* (rifampicin); the NBFE-164 strain harbored other ARGs of *aac(6')-Iaa*, *aadA2,* and *ant(3'')-Ia* (aminoglycoside), *bla*_TEM-1B_ (β-lactams), *cmlA1* and *floR* (phenicol), *dfrA12* (trimethoprim), *qnrS1* (quinolone), *sul2* and *sul3* (sulfonamide), and *tet(A*) and *tet(M)* (tetracycline).

### Genetic analysis of plasmid-carrying *bla*_NDM_ gene

**Fig 1:**
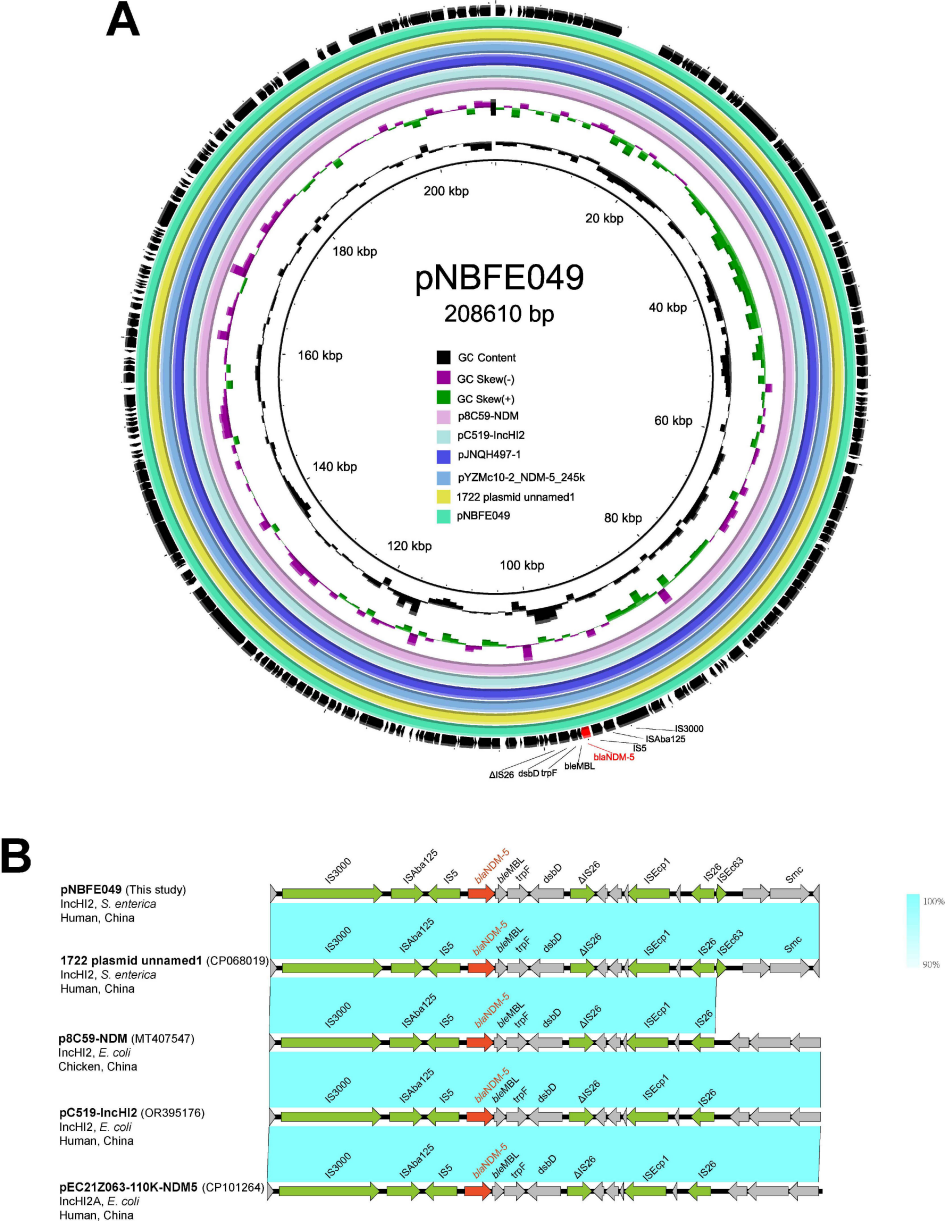
Genetic maps of the *bla*_NDM-5_-bearing plasmid in this study. (A) Comparative analysis of the *bla*_NDM-5_-bearing plasmid recovered from *S. enterica* NBFE-049 (IncHI2) with similar plasmids in the NCBI pathogen database. The outermost circle shows the open reading frames of pNBFE-049. (B) The genetic context of *bla*_NDM-5_ on the plasmid pNBFE-049 (this study) and the other four closely related plasmids retrieved from the NCBI pathogen database. The red arrow represents *bla*_NDM_, the green arrows represent Insertion Sequence (IS) elements, and the grey arrows represent additional coding sequences. The arrow’s direction indicates the direction of transcription. The homogeneous regions are represented by blue shadows.

Further findings showed that the gene *bla*_NDM-5_ in the NBFE-049 strain was located in a 208,610 bp IncHI2-type plasmid, named pNBFE-049. Except for *bla*_NDM-5_*,* pNBFE-049 also harbored ARGs of *aac(6')-Ib-cr*, *bla*_OXA-1_, *catB3*, *ARR-3*, and *sul1*. Blast analysis of pNBFE-049 found 100% coverage and 100% identity to the *S. enterica* strain 1722 NDM-5-positive plasmid, which was collected in November 2020 (CP068019). It also had high similarity to the plasmids in other species, i.e., *Escherichia coli* plasmid p8C59-NDM (MT407547), pC519-IncHI2 (OR395176), pJNQH497-1 (CP091926), and pYZMc10-2_NDM-5_245 k (CP123248) (Fig. 1A). Genetic environment analysis revealed that IS*3000*, IS*Aba125*, and IS*5* exist upstream of the *bla*_NDM-5_ gene, and truncated IS*26*, IS*Ecp1*, IS*26*, and IS*EC63* are located downstream (Fig. 1B). Together, the genetic structure of *bla*_NDM-5_ in the NBFE-049 strain was “IS*3000*-IS*Aba125*-IS*5-bla*_NDM-5_-*ble*_MBL_-*trpF-dsbD*-ΔIS*26*”, which also existed in the NDM-5-positive plasmid of 1722 strain (CP068019), p8C59-NDM (MT407547), pC519-IncHI2 (OR395176), and pEC21Z063-110K-NDM-5 (CP101264).

**Fig 2:**
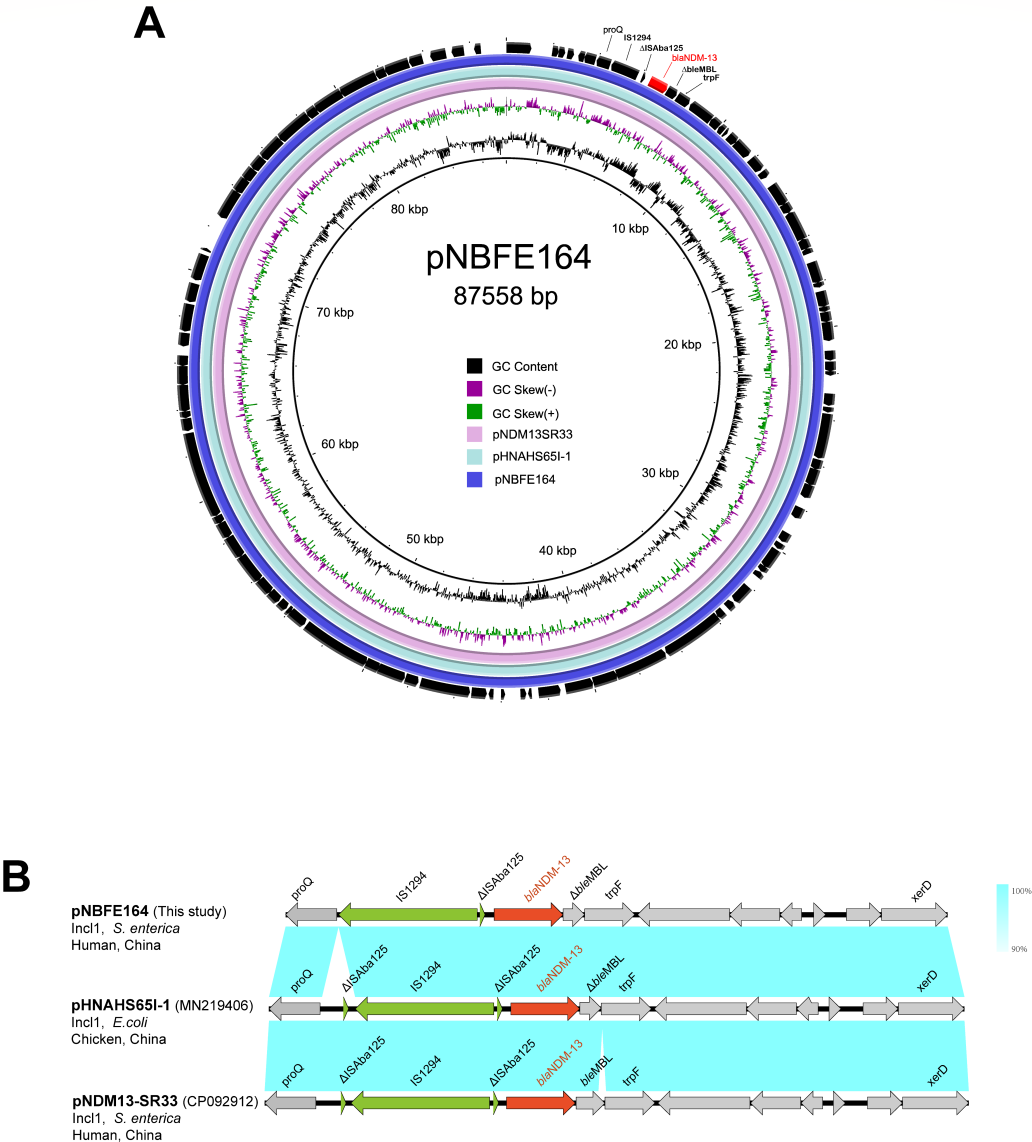
Genetic maps of the *bla*_NDM-13_-bearing plasmids in this study. (A) Comparative analysis of the *bla*_NDM-13_-bearing plasmid recovered from *S. enterica* NBFE-164 (IncI1) with similar plasmids in the NCBI pathogen database. The outermost circle shows the open reading frames of pNBFE-164. (B) The genetic context of *bla*_NDM-13_ on the plasmid pNBFE-164 (this study) and two other closely associated plasmids retrieved from the NCBI pathogen database. The red arrow represents *bla*_NDM_, the green arrows represent Insertion Sequence (IS) elements, and the grey arrows represent additional coding sequences. The arrow’s direction indicates the direction of transcription. The homogeneous regions are represented by blue shadows.

Besides, the carbapenemase gene *bla*_NDM-13_ in the NBFE-164 strain was located in an 87,558 bp IncI1-type plasmid, named pNBFE-164, with a G + C content of 50.4%. pNBFE-164 showed 100% coverage and 99.99% identity to *Salmonella* Rissen plasmid pNDM13-SR33 discovered in 2021 (CP092912.1) and showed high similarity to *E. coli* plasmid pHNAHS65I-1 (MN219406) as well (Fig. 2A). Furthermore, the genetic context of *bla*_NDM-13_ was similar among them, indicating that *bla*_NDM-13_ was located in a conserved genetic structure “IS*1294*-ΔIS*Aba125-bla*_NDM-13_-*ble*_MBL_-*trpF*” (Fig. 2B).

### Conjugation capabilities

The pNBFE-164 was readily able to conjugate from strain NBFE-164 to the recipient strain *E. coli* J53 with a high efficiency of 1.1 × 10^−2^. The transconjugant NBFE-164-J53 obtained resistant phenotypes to ampicillin, carbapenems (ertapenem and imipenem), cephalosporins (ceftriaxone, cefepime, and ceftazidime), and β-lactam/β-lactamase inhibitors (SAM and TZP) (Table 1). However, no transconjugants were obtained in the pNBFE-049 conjugation assay.

### Phylogenomic relationship

**Fig 3:**
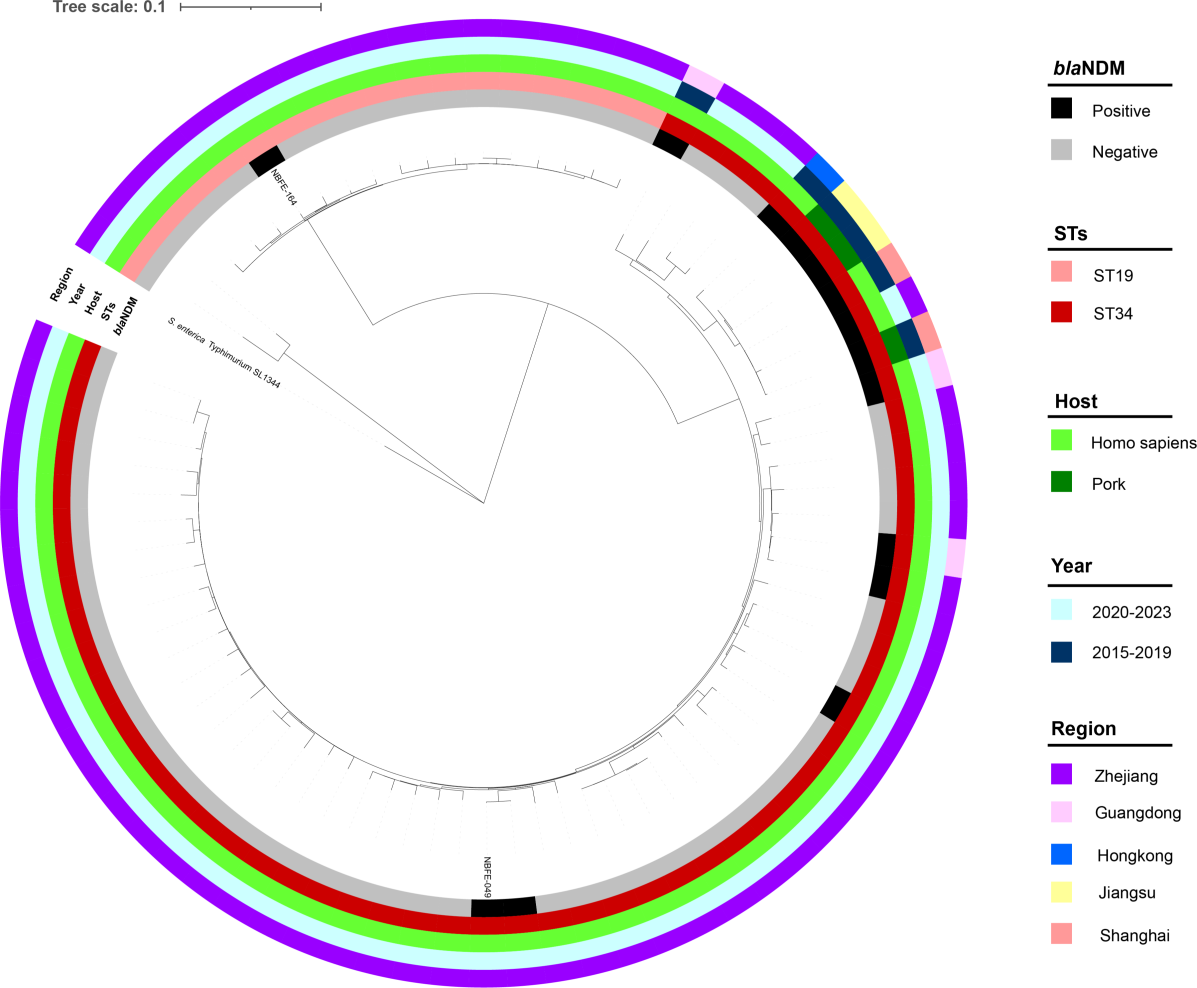
Phylogenetic analysis between NBFE-049, NBFE-164, SL1344, 12 isolates of NDM-producing *S*. Typhimurium (ST19 and ST34) from the NCBI database, and other *S*. Typhimurium (ST19 and ST34) in this area. The outgroup control strain was *S*. Typhimurium SL1344. The *bla*_NDM_-carrying status, sequence type, host, isolation time, and region (province/municipality directly under the central government) are represented by squares of different colors.

Twelve isolates of NDM-producing *S*. Typhimurium ST34 were obtained from the NCBI pathogen database, but no NDM-producing *S*. Typhimurium ST19 has been found (Table 2). The NBFE-049 strain showed only three SNP differences compared to the clinical strain 1722 (CP068018.1), which was isolated from a fecal sample of an infant in Dongyang, Zhejiang province, in November 2020. However, the SNPs between the NBFE-049 and the other local *S*. Typhimurium ST34 strains were more than 50 (50–129). It was also found that three pork-sourced *bla*_NDM-5_-positive *S*. Typhimurium ST34 strains SH160, W131, and YZU0489 (CP053294.1, GCA_027904115.1, and DATSRR000000000.1), which was isolated in Shanghai and Yangzhou, China, had <10 SNP differences from a strain SSH006 (MTKV00000000.1) of human origin in Shanghai. Additionally, the NBFE-164 strain had close genetic relationships to other local *S*. Typhimurium ST19 strains, with SNPs between 20 and 55 (Fig. 3).

**TABLE 2:** Summarizeder author information of 12 NDM-producing *S*. Typhimurium ST34 isolates obtained from the NCBI pathogen database

Strain	Host	Carbapenem-resistant gene	Year	Region	Bioproject	Biosample	GenBank	Serovar	ST
sg1722-2	Homo sapiens	*bla*_NDM-1_	2021	China, Zhejiang	PRJNA753290	SAMN20693596	CP081189.2	Typhimurium monophasic variant (1,4,[5],12:i:-)	34
81741	Homo sapiens	*bla*_NDM-5_	2015	China, Guangdong	PRJNA358799	SAMN06185755	CP019442.1	Typhimurium monophasic variant (1,4,[5],12:i:-)	34
3018683606	Homo sapiens	*bla*_NDM-5_	2021	China, Guangdong	PRJNA818143	SAMN26818630	CP094332.1	Typhimurium monophasic variant (1,4,[5],12:i:-)	34
1722	Homo sapiens	*bla*_NDM-5_	2020	China, Zhejiang	PRJNA689414	SAMN17208412	CP068018.1	Typhimurium monophasic variant (1,4,[5],12:i:-)	34
SSH006	Homo sapiens	*bla*_NDM-5_	2015	China, Shanghai	PRJNA362548	SAMN06241972	MTKV00000000.1	Typhimurium monophasic variant (1,4,[5],12:i:-)	34
SH160	Minced pork	*bla*_NDM-5_	2016	China, Shanghai	PRJNA625025	SAMN14591152	CP053294.1	Typhimurium monophasic variant (1,4,[5],12:i:-)	34
Children	Homo sapiens	*bla*_NDM-1_	2019	China, Hong Kong	PRJNA678820	SAMN16824857	DAFIDB000000000.1	Typhimurium monophasic variant (1,4,[5],12:i:-)	34
1104–75	Homo sapiens	*bla*_NDM-5_	2021	China, Guangdong	PRJNA884865	SAMN31055796	CP110198.1	Typhimurium monophasic variant (1,4,[5],12:i:-)	34
S2122	Homo sapiens	*bla*_NDM-5_	2022	China, Zhejiang	PRJNA898106	SAMN31601029	CP110657.1	Typhimurium monophasic variant (1,4,[5],12:i:-)	34
W131	Pork	*bla*_NDM-5_	2016	China, Jiangsu	PRJEB33347	SAMEA5756187	DAMRYG000000000.1	Typhimurium monophasic variant (1,4,[5],12:i:-)	34
YZU0489	Pork	*bla*_NDM-5_	2016	China, Jiangsu	PRJEB50816	SAMEA114307272	DATSRR000000000.1	Typhimurium monophasic variant (1,4,[5],12:i:-)	34
2023J × 045	Homo sapiens	*bla*_NDM-5_	2023	China, Zhejiang	PRJNA1010012	SAMN37177606	DAQDXQ000000000.1	Typhimurium monophasic variant (1,4,[5],12:i:-)	34

## DISCUSSION

Two carbapenem-resistant NTS isolates were identified among 511 NTS strains, with a carbapenem-resistant rate of 0.4%. One (NBFE-049 strain) belonged to *S*. 1,4,[5],12:i:- ST34, and the other (NBFE-164 strain) was identified as *S*. Typhimurium ST19. These two isolates were resistant to most examined β-lactams. Luckily, they were sensitive to azithromycin, an alternative antibiotic for treating invasive or severe NTS infections. Although the Clinical and Laboratory Standards Institute (CLSI) has not established the interpretive standards of azithromycin for NTS, some studies suggest that the azithromycin-resistant MIC breakpoints of *S*. Typhi recommended by CLSI can be expanded to NTS (24, 25). The two children underwent empirical antimicrobial therapy with cephalosporins (latamoxef, ceftriaxone, or ceftazidime) first, but symptoms persisted. Then, they were administered azithromycin after obtaining antibiotic susceptibility tests, and both recovered, which also highlights the importance of antimicrobial susceptibility tests.

By whole genome sequencing (WGS), genes *bla*_NDM-5_ and *bla*_NDM-13_ were identified as the causes for their carbapenem resistance. Gene *bla*_NDM-5_ in the NBFE-049 strain was carried by a IncHI2-type plasmid, pNBFE-049, which also carried other ARGs of *aac(6')-Ib-cr*, *bla_OXA-1_*, *catB3*, *ARR-3*, and *sul1*. The *bla*_NDM-5_-carrying plasmids in *S*. Typhimurium isolates mainly belong to IncX3 and IncFII replicon types (20–22, 26, 27), and IncHI2-plasmid carrying *bla*_NDM-5_ in clinical *S*. Typhimurium isolates is rarely reported. Although several mobile elements were located upstream and downstream of pNBFE-049, no transconjugants were obtained in the conjugation assay, which was repeated three times under our conjugation experimental conditions.

Plasmid pNBFE-164, an IncI1-type plasmid in the NBFE-164 strain, harbored a carbapenem resistance gene of *bla*_NDM-13_, which was located in a conserved genetic structure “IS*1294*-ΔIS*Aba125-bla*_NDM-13_-*ble*_MBL_-*trpF*”. Similar genetic structures were also presented in the pHNAHS65I-1 (MN219406) and pNDM13-SR33 (CP092912.1). The major difference was that pNBFE-164 lacked a truncated IS*Aba125* element located upstream, indicating the diversity of the IncI1 plasmid carrying *bla*_NDM-13_. Besides, the conjugation assay showed that pNBFE-164 could be transferred to the recipient strain with high efficiency. Plasmid pHNAHS65I-1 from *E. coli* and pNDM13-SR33 from *S*. Rissen were also IncI1 plasmids. Although it was unknown whether pHNAHS65I-1 could be transferred, pNDM13-SR33 from *S*. Rissen was reported as a transmissible plasmid (28). These findings suggested that the IncI1 plasmid may serve as a critical vector to spread *bla*_NDM-13_.

Finally, through phylogenomic analysis, the NBFE-049 strain showed only three SNP differences from strain 1722 (CP068018.1), but more than 50 SNP differences from the other local *S*. Typhimurium ST34 strains. Strain 1722 was isolated from an infant in Dongyang, Zhejiang province, in November 2020. The few SNP differences and their temporal and spatial proximities suggested they had a close genetic relationship. Combined with the fact that pNBFE-049 cannot be transferred by conjugation assay, the *bla*_NDM-5_-positive strain NBFE-049 may have originated from clonal spread. Similarly, we also found a *bla*_NDM-5_-positive *S*. Typhimurium ST34 strain of human origin that was closely related to three pork-sourced strains isolated in the same region or nearby, suggesting *S*. Typhimurium ST34 producing NDM-5 has the potential to be clonally transmitted through the pork supply chain. Therefore, the clone spread of *bla*_NDM-5_-positive *S*. Typhimurium ST34 should be vigilantly monitored.

As there was no available dataset of NDM-positive *S*. Typhimurium ST19 in the NCBI database, we only compared the SNP differences of the NBFE-164 strain to other local *S*. Typhimurium ST19 strains. It showed the NBFE-164 strain had a close genetic relationship to other local *S*. Typhimurium ST19 strains, as the SNPs between them ranged from 20 to 55. With the result that pNBFE-164 was readily able to conjugate, *bla*_NDM-13_ in the NBFE-164 strain may be due to plasmid diffusion.

### Conclusion

Overall, we reported the genomic characteristics of two *S*. Typhimurium strains carrying *bla*_NDM-5_ and *bla*_NDM-13_, which provided new insights into the antimicrobial resistance mechanism and transmission risk of *bla*_NDM_-positive NTS in China. We suggested the potential contributions of clonal spread and plasmid-mediated *bla*_NDM_ transfer in CRSE dissemination. Future enhanced surveillance policy should mitigate CRSE spreading, and more importantly, clinical antimicrobial therapeutic regimens should be adjusted accordingly.

## MATERIALS AND METHODS

### Bacterial collection

In total, 511 non-redundant NTS clinical isolates were recovered from children admitted to the Women and Children’s Hospital of Ningbo University between January 2014 and December 2023. Two strains of carbapenem-resistant *Salmonella* (NBFE-049 and NBFE-164) were collected, with both resistant to imipenem and ertapenem. The NBFE-049 strain was isolated from a boy hospitalized for fever and convulsion in June 2021, while the NBFE-164 strain was recovered from a girl in January 2023, who was admitted for treating diarrhea and fever. The bacterial species were confirmed by matrix-assisted laser desorption ionization–time of flight mass spectrometry (bioMérieux, France).

Clinical data were collected from their electronic medical records, and the two children recovered after administration of cephalosporins and azithromycin. This study was approved by the institutional ethics committee (EC2022-022).

### Antimicrobial susceptibility tests

The antibiotic susceptibility tests were performed using the VITEK 2 COMPACT automatic analysis system (bioMérieux, France) employing an AST GN13 panel, including ertapenem, imipenem, levofloxacin, ciprofloxacin, aztreonam, cefepime, ceftriaxone, ceftazidime, TMP-SMX, TZP, SAM, and ampicillin. Azithromycin MICs were obtained by E-test (Bio-kont, China), and the MICs were read at 100% inhibition. The susceptibility breakpoints were those recommended by the CLSI guidelines (34th Edition).

### Whole genome sequencing (WGS) and bioinformatic analysis

Genomic DNA was extracted using a TIANamp Bacteria DNA kit (TIANGEN Biotech, China). The WGS was performed using the Nanopore MinlON Platform (Nanopore, Oxford, UK) and Illumina NovaSeq 6000 Platform (Illumina Inc., San Diego, U.S.A). Complete genome sequences were annotated using Unicycler (v0.4.5) (29). Then, the *in silico* serotyping was predicted using the *Salmonella In Silico* Typing Resource (https://github.com/phac-nml/sistr_cmd). The STs, ARGs, and plasmid replicons were detected using the assemblies of the samples on the in-house Galaxy platform in combination with MLST v2.22.0 (https://github.com/tseemann/mlst), and Abricate v1.0.1 (https://github.com/tseemann/abricate), including ResFinder database, and PlasmidFinder database. Comparisons of *bla*_NDM_ harboring plasmids with their associated plasmids were performed using BLASTn v2.4.0 (30) and visualized using Easyfig v2.2.3 (31), and BLAST Ring Image Generator.

### Plasmid conjugation experiment

Plasmid conjugation experiments were performed on the NBFE-049 and NBFE-164 isolates as described previously (32). A sodium azide-resistant *E. coli* strain J53 was used as the recipient strain. Fresh cultures of the donor and recipient strains were adjusted in concentration and co-incubated in a ratio of 1:1 of donor to recipient cells on the Luria-Bertani agar plate at 37°C overnight. Transconjugants were selected on Luria-Bertani agar plates containing sodium azide (100 µg/mL) and meropenem (2 µg/mL). The number of transconjugants and recipients was recorded, which was used to calculate the transfer frequency. The antimicrobial susceptibility of the transconjugant was assessed using the VITEK 2 COMPACT automatic analysis system (bioMérieux, France) employing an AST GN13 panel.

### Phylogenomic analysis

Based on the genomes of 12 isolates of NDM-producing *S*. Typhimurium ST34 (obtained from the NCBI pathogen database until March 18, 2024) and 63 carbapenem-sensitive *S*. Typhimurium ST19 and ST34 strains (isolated in a local context during the studied period), a phylogenomic analysis was performed to investigate the origin and evolution of the NBFE-049, and NBFE-164 strains. A core-genome SNP-based phylogenetic tree was built using the Harvest suite (33). After removing genomic sequences in the recombination regions, Parsn was used to calculate pairwise SNPs. A maximum-likelihood phylogenetic tree with 1,000 bootstraps was generated using IQ-TREE v1.6.1. The phylogenetic tree and associated data were visualized with iTOL v6.9 (34).

## Data Availability

All the sequencing data of the NTS isolates from this study are deposited in Sequence Read Achieve under BioProject numbers PRJNA998333 and PRJNA1097312. The complete genome sequences of strains NBFE-049 and NBFE-164 have been uploaded to GenBank under the accession numbers JBEFON000000000 and JBEFNU000000000, respectively.
